# Current Strategies to Optimize Nutrition and Growth in Newborns and Infants with Congenital Heart Disease: A Narrative Review

**DOI:** 10.3390/jcm11071841

**Published:** 2022-03-26

**Authors:** Guglielmo Salvatori, Domenico Umberto De Rose, Anna Claudia Massolo, Neil Patel, Irma Capolupo, Paola Giliberti, Melania Evangelisti, Pasquale Parisi, Alessandra Toscano, Andrea Dotta, Giovanni Di Nardo

**Affiliations:** 1 Neonatal Intensive Care Unit, Medical and Surgical Department of Fetus-Newborn-Infant, “Bambino Gesù” Children’s Hospital, IRCCS, 00165 Rome, Italy; domenico.derose@opbg.net (D.U.D.R.); ac.massolo@gmail.com (A.C.M.); irma.capolupo@opbg.net (I.C.); paola.giliberti@opbg.net (P.G.); andrea.dotta@opbg.net (A.D.); 2 Human Milk Bank, Medical and Surgical Department of Fetus-Newborn-Infant, “Bambino Gesù” Children’s Hospital, IRCCS, 00165 Rome, Italy; 3 PhD Course in Microbiology, Immunology, Infectious Diseases, and Transplants (MIMIT), Faculty of Medicine and Surgery, University of Rome “Tor Vergata”, 00133 Rome, Italy; 4 Department of Neonatology, Royal Hospital for Children, Glasgow G51 4TF, UK; neilpatel1@nhs.net; 5 NESMOS Department, Chair of Pediatrics, Faculty of Medicine and Psychology, Sapienza University of Rome, Sant’Andrea University Hospital, 00189 Rome, Italy; melania.evangelisti@uniroma1.it (M.E.); pasquale.parisi@uniroma1.it (P.P.); giovanni.dinardo@uniroma1.it (G.D.N.); 6 Perinatal Cardiology Unit, Medical and Surgical Department of Fetus-Newborn-Infant, “Bambino Gesù” Children’s Hospital, IRCCS, 00165 Rome, Italy; alessandra.toscano@opbg.net

**Keywords:** congenital heart disease, nutrition, growth, necrotizing enterocolitis, parenteral nutrition, enteral feed

## Abstract

(1) Objective: This review aims to identify the clinical and practical barriers to optimizing nutrition in newborn infants with congenital heart disease (CHD) and to describe updated evidence-based recommendations for clinical and nutritional management of these patients in a narrative review. (2) Research Methods and Procedures: We conducted a search of the relevant literature published from 2000 to December 2021. (3) Results: CHD patients undergo several nutritional challenges related to the underlying cardiac disease anomaly, the potential increased risk of NEC, and delayed enteral feeding, resulting in inadequate energy intake and sub-optimal growth, increased morbidity and mortality. (4) Conclusions: To optimize nutrition and growth in newborn infants with CHD, standardized protocols should be implemented. Regular nutritional and growth assessment with a multi-disciplinary team is essential. We propose a decisional algorithm that may represent a potentially useful tool to guide clinicians to optimize growth and nutrition.

## 1. Introduction

Congenital heart disease (CHD) is the most common birth defect with a prevalence of 9 per 1000 live births [1]. Disease severity and treatment choice depend on the underlying anomaly. Despite advances in medical and surgical management leading to more affected children now reaching adulthood, intestinal dysfunction, poor nutrition, and growth failure remain common in infants with CHD [2,3]. Increased metabolic demand, reduced calorie intake, malabsorption, genetic factors, and fluid restriction may result in an energy imbalance that negatively affects morbidity and mortality in these patients [4,5]. Malnutrition is a significant risk factor for adverse post-surgical outcomes [6].

Improved nutrition may be fundamental to stimulating growth, wound healing, myocardial and vascular function, reducing the length of hospital stay, the risk of nosocomial infections, and improving neurodevelopmental outcomes [7,8]. Beyond the type of heart defect, if infants with CHD are born prematurely, then they have higher odds for mortality than their peers without congenital anomalies. If the CHD is severe, in-hospital mortality increases two to three times [9]. Furthermore, extra-uterine growth restriction (EUGR) is common in preterm infants even in the absence of CHD and has been associated with a poor neurodevelopmental outcome [10].

However, addressing these challenges in newborn infants with CHD has, in part, been limited by the absence of guidelines and standardized approaches for clinical assessment and treatment. In 2009, the American Society of Parenteral and Enteral Nutrition recommended standard feeding protocols for pediatric patients in the Intensive Care Unit (ICU) for improved nutritional care and growth in critically ill patients, particularly those with CHD [11].

This review aims to critically appraise currently described nutrition care in CHD patients including assessing their practical utility and limitations. We then describe updated evidence-based recommendations for future clinical/nutritional management of these patients.

## 2. Materials and Methods

A literature search was performed using PubMed (https://pubmed.ncbi.nlm.nih.gov/, accessed on 26 January 2022), eMedicine Medscape (https://emedicine.medscape.com/, accessed on 26 January 2022), Scopus (https://www.scopus.com/, accessed on 26 January 2022) and Ovid MEDLINE (https://www.wolterskluwer.com/en/solutions/ovid/ovid-medline-901, accessed on 26 January 2022). Search terms were: “parenteral nutrition” AND “enteral nutrition” AND “infants” OR “neonates” OR “newborn” AND “congenital heart defects” OR “congenital heart disease”. Studies published from January 2000 to December 2021 were selected in order to include all studies in the modern treatment era. We focused on articles published in the last 5 years, when possible.

Abstracts and full papers were reviewed by two authors (ACM and GS) and selected based on their relevance to CHD and nutrition. In addition, reference lists from papers that met the criteria, together with current pediatric CHD guidelines, were reviewed by a third reviewer (DUDR) to identify any additional relevant papers.

## 3. Nutritional Challenges in CHD Patients

The increased metabolic demand in CHD is attributed to the combination of chronic hypoxia, increased cardiorespiratory work, venous congestion, increased pulmonary artery pressures, and catecholamine secretion [8,12]. Ventricular hypertrophy and dilation, which are frequently observed in these patients, increase myocardial oxygen consumption to 20% to 30% of the body’s total oxygen consumption, instead of the typical 10% [13]. The concomitant metabolic requirements for growth, cognitive and motor development are also necessary [8]. 

Adequate energy supply is a key factor to help children reach optimal clinical conditions before surgery, resulting in a decreased risk of morbidity and mortality. The preoperative poor nutrition of children with CHD impacts the postoperative rehabilitation process, contributing to delayed healing of the surgical wound, myocardial dysfunction, endothelial damage, reduced muscle function and increased risk of post-operative infections (in particular pneumonia) [14,15,16]. 

As a result, patients with hemodynamically significant CHD require increased nutritional support compared to healthy infants. Energy intake need may vary from 130–150 kcal/kg/day to 175–180 kcal/kg/day depending on the type of CHD [13].

### 3.1. CHD Hemodynamics and the Consequences for Nutrition

**Figure 1:**
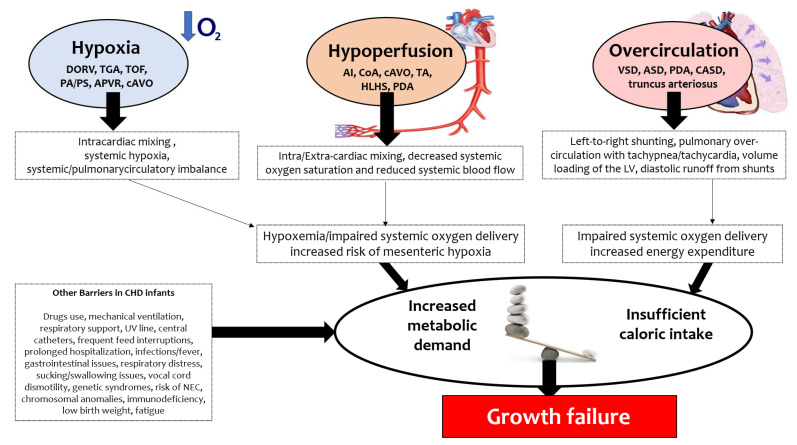
Growth failure mechanisms in congenital heart disease.

Three hemodynamics mechanisms influence nutrition and growth failure (shown in Figure 1):

(1) **Hypoxia**, such as in double-outlet right ventricle (DORV), transposition of the great arteries (TGA), tetralogy of Fallot (TOF), pulmonary atresia/pulmonary stenosis (PA/PS), anomalous pulmonary venous return (APVR), critical aortic valve obstruction (cAVO);
(2) **Hypoperfusion**, such as in aortic interruption (AI), aortic coarctation (CoA), cAVO, tricuspid atresia (TA), hypoplastic left heart syndrome (HLHS), patent ductus arteriosus (PDA);
(3) **Overcirculation**, such as in ventricular septal defect (VSD), atrial septal defect (ASD), PDA, complete atrioventricular septal defect (CASD), truncus arteriosus.

Malnutrition is common in neonates with cyanotic CHD, particularly if associated with pulmonary hypertension [13,17]. These are associated with right-to-left shunting, resulting in hypoxemia leading to weight and height impairment. These defects, which are characterized by an intracardiac mixing, require a delicate balance to provide adequate pulmonary and systemic blood flow. Circulatory imbalance may also lead to decreased mesenteric blood flow, resulting in malabsorption, feeding intolerance or intestinal ischemia [13,18]. For example, when repair is postponed for various reasons (such in the case of infants with tetralogy of Fallot (TOF) from developing countries who did not undergo primary complete repair in infancy), children are more hypoxemic and growth-restricted, and have a worse left ventricular ejection fraction [19]. Conversely, early repair of TOF results in significant acceleration of weight and height and restoration of genetic growth potential [20].

Acyanotic CHD, such as large left-to-right shunts, may be associated with pulmonary overcirculation, characterized by tachypnoea, hepatomegaly, and tachycardia, leading to increased metabolic demand, which combined with the use of diuretics, may result in growth failure [13]. The growth improvement with increasing age could be, in some cases, due to a decrease in the size of a left-to-right shunt consequent to either spontaneous decrease or closure of a septal defect or the onset a pulmonary vascular obstructive disease, highlighting the role of cardiac shunts in affecting growth [21].

CHD characterized by an obstruction to systemic blood flow, such as left ventricular outflow obstruction and aortic arch anomalies, are associated with intra and extra-cardiac mixing, resulting in decreased systemic oxygen saturation and reduced systemic blood flow. The resulting impaired systemic oxygen delivery leads to acute end-organ dysfunction, including compromised mesenteric oxygenation [18].

Furthermore, CHD patients are more often exposed to the use of multiple drugs and medications, such as inotropes, which may themselves increase myocardial oxygen and energy demand. Analgesia, sedation, and muscle relaxation used in critical care may delay oral and enteral feeding. Unstable CHD patients are exposed to prolonged duration of mechanical ventilation, or prostaglandin E1 (PGE1) infusion, which may also contribute to delays in optimal enteral feeding [8,22]. 

Although a higher risk of mesenteric arterial thrombosis has been postulated when an umbilical arterial catheter (UAC) is in place, due to disruption of the blood flow [23], the ESPNIC (European Society of Pediatric and Neonatal Intensive Care) issued a policy statement in favor of the introduction of enteral feeding in term neonates with CHD [24]. 

When ductal patency should be maintained, such as in obstruction to systemic circulation, obstruction to pulmonary circulation, and inadequate mixing of pulmonary and systemic blood flow, PGE1 infusion is paramount. According to a recent European survey, infants receiving PGE1 infusion were routinely fed in only 63% of the responding units [16]. Nordenström et al. recently noted a low risk of necrotizing enterocolitis in enterally-fed neonates with critical CHD, even in ductal-dependent systemic circulation [25]. Given that a low-dose PGE1 infusion (0.01 µg/kg/min) has been shown to be a safe practice in these infants [26] and even long-term infusion have relatively minor side effects [27], a priori pre-operative enteral fasting during PGE1 administration should be discouraged and individualized. 

### 3.2. Prematurity, Low Birthweight and Genetic Anomalies

Growth restriction may be proportional to the degree of heart failure and/or hypoxia, rather than uniformly occurring if an infant has a mild cyanosis or a small defect, unless other factors affect this outcome, such as prematurity and genetic or syndromic disorders. 

Prematurity and low birthweight add to the risk of serious CHD in infants, with a new definition of “low birth weight” for cardiac surgery moving toward 2 kg. Anderson et al. assessed that cardiac surgery increased the risk of mortality from 4.8% to 9.5% in neonates with a weight less than 2 kg [28]. Furthermore, growth is poorer in preterm infants with CHD than their peers without, although the reported incidence of growth restriction can vary widely in preterm infants, according to the different definitions and growth charts that are used. Growth should be longitudinally monitored once a week in preterm infants, after the physiological loss of fluids in the first weeks of life, in order to make changes in the nutritional strategy and promptly intervene while infants are still hospitalized [10], and thus beyond the type of heart defect.

Overall, approximately up to 30% of CHD are due to known chromosomal, genetic or other anomalies that can be associated with growth restrictions, with a higher incidence of small for gestational age (SGA) infants when CHD is not isolated, such as in the case of Down syndrome or Turner syndrome. The increased rate of SGA infants may also be due to maternal, fetal or placental components that can influence both CHD and growth restriction since fetal life. [29]. 

Moreover, anomalies in fetal hemodynamics and oxygen saturation due to CHD play a key role. Beyond the presence or not of a CHD, growth-restricted preterm infants have a maladaptive arterial–ventricular coupling [30]. All these aspects concur afterwards in growth-restriction of preterm CHD infants: for example, when comparing catch-up growth in term and preterm infants after surgical repair of ventricular septal defect, preterms caught up later than term infants [31].

### 3.3. Other Clinical Barriers

Enteral feeding is often temporarily discontinued, reduced, or postponed, including for surgical procedures. The reasons for interrupting enteral feeding include deterioration in the clinical status, extubation failure and need of mechanical ventilation or respiratory support, placement of chest tubes or central venous catheters, infections/fever, various gastrointestinal issues (such as gastro-esophageal reflux, feed intolerance or emesis, abdominal distention, or presence of bloody stools), sucking/swallowing issues, chronic fatigue, and vocal cord dysmotility. Genetic syndromes, additional non-cardiac anomalies and unknown causes may also influence enteral feeding [5,6,18,22].

Postoperatively, extracardiac complications (e.g., chylothorax, infections, acute kidney injury) can occur, necessitating withholding of enteral feeds [22]. Among critical CHD, infants with hypoplastic left heart syndrome (HLHS) are more likely to undergo several surgical treatments and are therefore at even greater risk of failure to thrive [32]. 

Nutrition and growth after surgery are affected by poor preoperative nutritional state, combined with a complex inflammatory state, and protein catabolism [5,15].

The prolonged hospitalization that often characterizes these patients, may expose infants to repeated infections and fever, further impairing feeding, optimal nutrition, and caloric expenditure [13,33].

### 3.4. Necrotizing Enterocolitis: Risks and Fear

Prevention and management of necrotizing enterocolitis (NEC) requires special consideration in infants with CHD. The incidence of NEC in neonates with CHD ranges from 2% to 8% and may increase up to 20%, particularly in those with a single ventricle physiology and ductal dependency (such as HLHS) [8]. NEC usually develops earlier in CHD infants than is typically observed in preterm infants. Furthermore, in the CHD population, the colon is more often involved compared to the small intestine or ileocecal region in the preterm population [34].

The pathophysiological mechanisms underlying NEC in CHD infants may be different to those in preterm infants. Contributing factors include impaired mesenteric blood flow due to low cardiac output, restricted flow in duct-dependent lesions, and diastolic run-off from shunts, as well as systemic hypoxia and acidosis associated with cyanotic defects as well as rapid increases in feed volume [13,25]. Other risk factors associated with increased risk of NEC in CHD infants include chromosomal anomalies, immunodeficiency, and low birth weight [25].

For these reasons, clinicians may have concerns about initiating and increasing feeds in CHD patients, leading to frequent interruption or delay, which may itself be deleterious [25]. Prolonged periods of fasting result in intestinal villus atrophy as well as intestinal microbiota impairment with loss of barrier function, which may increase the risk for NEC and negatively affect growth [22,35,36].

Early commencement of minimal enteral feeding (MEF) has been shown to improve the development of the intestinal mucosa and maturation of gut immune response [33]. An exclusive human milk diet may reduce the risk of pre-operative NEC in infants with CHD [18,36,37]. Additionally, the use of probiotics in newborns with ductal-dependent CHD has been potentially associated with reduced risk of NEC [38].

Conversely, caution should be used in fortifying feeds in premature infants with serious CHD, considering the increased post-fortification osmolality of human milk, which should be measured before administration to high-risk infants (if possible) [39]. 

During enteral feeding, CHD patients should be monitored closely for early signs of NEC (Table 1). Near-infrared spectroscopy (NIRS) and mesenteric blood flow velocities may also assist in monitoring gut oxygenation, to guide clinical management including potential early detection of mesenteric ischemia [40,41,42]. Furthermore, intestinal ultrasound markers (pneumatosis intestinalis, portal venous gases, bowel wall thinning or thickening, peritoneal fluid, absent bowel wall signature, hyperaemia by colour-Doppler, fixed dilated bowel loops) can provide a more accurate evaluation of gut injury and diagnosis of NEC, in comparison to standard abdominal X-ray [43]. Guidelines and standardized recommendations in preterm infants have reduced the incidence of NEC [44]. However, the current lack of standardized protocols in the preoperative enteral nutrition management of infants with CHD leads to clinician variability on when, with what and how to feed. This potentially exposes new-born infants to negative post-operative outcomes such as NEC, poor growth and increased length of hospital stay [35].

## 4. Pre- and Post-Operative Strategies and Recommendations

**Figure 2:**
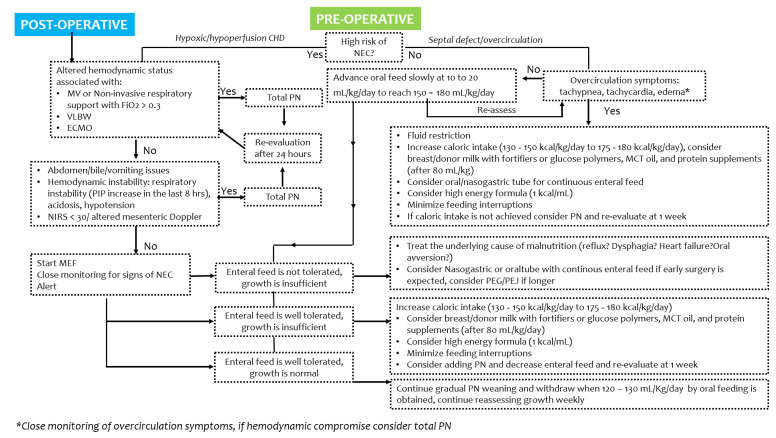
Proposed algorithm to optimize nutrition in newborn infants with congenital heart diseases. MV, mechanical ventilation; VLBW, very low birth weight; PIP, positive inspiratory pressure; MEF, minimal enteral feeding; PN, parenteral nutrition.

**Figure 3:**
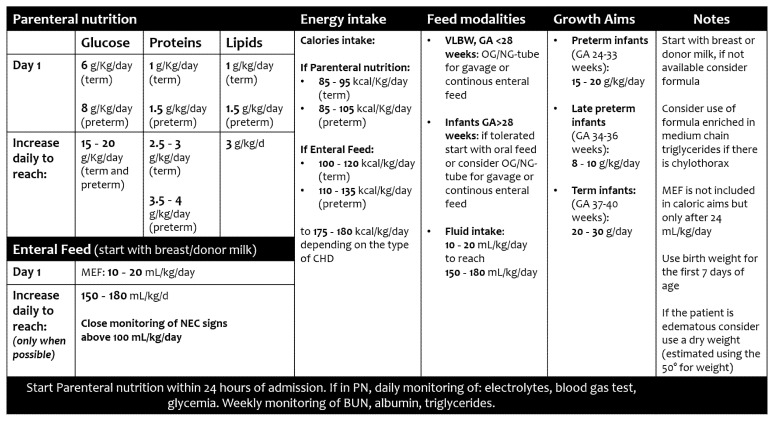
Target energy intake and growth aims in newborn infants with congenital heart diseases. GA, gestational age; OG, orogastric; MEF, minimal enteral feeding; NEC, necrotizing enterocolitis; NG, nasogastric.

Adequate nutrition is crucial in children with CHD; a structured approach to correct timing and type of feeding may be beneficial. Strategies to optimize nutrition in CHD cases may be challenging, depending on the infants’ age, or the congenital heart defect, and the timing (pre- or post-operative period), though the goal remains to increase the quality or volume of feeds and to support the metabolic demands (Figure 2 and Figure 3) [13,14,16].

In centers where feeding protocols have been developed for infants with CHD, the observed benefits include improved postoperative enteral feeds, reduced parenteral nutrition (PN) use, fewer placements of nasogastric and gastric tubes, and improved weight and outcomes such as NEC onset, hospital stay and mortality [8,45,46,47,48]. 

### 4.1. Total Nutritional Requirements

#### 4.1.1. Fluid Volume

The adequate total amount of fluid required is calculated depending on the cardio-respiratory condition at birth and in the early days of life. 

Fluid volume should then enable catch-up growth without promoting overcirculation or altering the fluid retention [13]. In infants with CHD characterized by pulmonary over-circulation, fluid restriction or/and use of diuretics are key symptom-management strategies [49]. Conversely, fluid restriction is reported to be a barrier in providing adequate nutrition and may exacerbate poor pre-operative nutritional status, further reducing limited energy and protein reserves [49]. 

Increasing the quality and quantity of dietary nutrients, and focusing on nutrient energy-dense feed, are associated with improved weight gain and the achievement of nutritional targets [1,2]. Total fluid intake in conjunction with optimal energy protein supply, guided by a multidisciplinary team is essential [50,51].

Fluid overload has been related to adverse outcomes, such as acute kidney injury, more days on mechanical ventilation, greater need of vasoactive drugs, delayed chest closure after neonatal cardiac repairs, and mortality [52].

In the preoperative period, clinicians should consider the changes in pulmonary vascular resistance and the possible risk of excessive pulmonary blood flow, especially in those who receive PGE1 infusion. Conversely, relative hypovolemia could also occur given the insensible losses due to enteral fasting before surgery and tachypnoea [53].

During surgical repair, the use of cardiopulmonary bypass (CPB) deeply influences fluid homeostasis, with haemodilution due to crystalloid prime, inflammatory reaction and capillary leak-syndrome after CPB leading to a significant risk of myocardial and pulmonary oedema [54]. 

The post-operative period is more often characterized by fluid accumulation than hypovolemia; therefore, according to a worldwide survey, most pediatric intensivists usually limit total fluid intake to 50% during the first 24 h after cardiac surgery, despite a lack of evidence on what extent of fluid restriction may be sufficient [55].

#### 4.1.2. Energy Intake

Indirect calorimetry (IC) appears to be the most accurate tool to quantify resting energy expenditure (REE) in CHD infants; it can distinguish hypometabolism (<90% of predicted), normal metabolism (90–110% predicted) and hypermetabolism (>110% predicted) patterns [56]. If IC measurement is not feasible, clinical equations (such as Schofield or WHO equations) may be used to estimate REE, although no presently used equation seems to precisely measure it, especially in infants under 5 kg and after CPB, up to a mean discrepancy of 16.9 kcal/kg/day [56,57]. 

However, there is a strong consensus (according to ESPNIC recommendations) that energy intake provided should not exceed REE in the acute phase, whereas afterwards, the energy intake provided to critically ill children should be personalized for energy debt, physical activity, rehabilitation, and growth [24].

#### 4.1.3. Protein Intake

Protein loss is frequently associated with CHD, particularly following surgical treatment [58]. A primary aim in infants with CHD is also to provide sufficient dietary protein to enable adequate new protein synthesis. Proteins are necessary for tissue repair and growth, and they facilitate wound healing, modulate inflammatory responses, and preserve skeletal muscle mass [8,51].

A minimum protein intake of 1.5 g/kg/day is recommended by both European and American guidelines, to prevent a cumulative negative protein balance [24,56]. A standard protein requirement of up to 3 g/kg/day should be ensured in critically ill infants aged <2 years [8,11,47], especially those requiring mechanical ventilation [56].

However, administration of protein via PN should be done with caution, particularly in critically ill patients. Findings obtained in cohorts of preterm infants showed that a high dose of aminoacids provided parenterally seems to affect the size of brain structures during neonatal life, whereas enteral protein intake is more likely associated with better brain development [59].

### 4.2. Parenteral Nutrition

To support the growth of infants with CHD, particularly when the enteral support exceeds the metabolic reserves, it is recommended that PN be started as soon as possible. This may concern cases in the early postoperative stage, or mechanically ventilated, or with gastrointestinal issues [13]. 

Use of intravenous intralipids in addition to enteral feeding was also suggested as a support to those children with insufficient enteral feeds, to prevent fatty acid deficiency [5,13]. Given that systemic inflammation may influence outcomes in CHD infants [60,61], the use of new multicomponent lipid emulsions (MLE) should be preferred; they are rich in ω-3 fatty acid metabolites (such as docosahexaenoic and eicosapentaenoic acids) that can stimulate anti-inflammatory pathways [62], compared to standard soybean lipid emulsions (SLE) that are only rich in ω-6 fatty acids [63]. This has been confirmed also in infants undergoing open-heart surgery [64]. Recently, concerns have been raised over the adverse effects of excessive PN. Withholding parenteral nutrition while administering micronutrients intravenously for a week in critically ill infants has been associated with fewer new infections, a shorter duration of dependency on ICU, and a shorter hospital stay [65].

### 4.3. Enteral Nutrition

#### 4.3.1. Types of Feed

Clinicians may be reluctant to initiate preoperative trophic feed in infants with CHD, due to fear of NEC [66]. However, early enteral feeding appears safe and is associated with a shorter duration of mechanical ventilation, a trend toward more stable postoperative hemodynamics, less fluid overload, and earlier postoperative feeding tolerance [67]. Minimal enteral feeding (MEF, defined as 10 to 20 mL/kg/day of milk) has been associated with improved intestinal mucosa development and reduced risk of NEC [8]. 

After surgical repair, infants usually tolerate trophic feeds; moreover, trophic feeds appear to decrease the infection-related morbidity following surgery [68]. Furthermore, the early initiation of feeding helps to meet energy targets in a shorter period [69]. 

Data obtained in preterm infants show how the presence of a patent ductus arteriosus (PDA), with either a pulsatile or restrictive shunt pattern and no evidence of reversed end-diastolic flow in descending aorta, does not seem to have a significant influence on splanchnic oxygenation in response to the first enteral feed [70]. Similarly, in CHD infants, the NIRS monitor is a feasible tool to easily monitor splanchnic oxygenation, which is well correlated with serum lactate and measurements of systemic mixed venous saturation (SVO2) [71].

Breast milk has been demonstrated to be protective in term, preterm and infants undergoing surgery and therefore should be encouraged in the CHD population [47,72,73]. Unfortunately, infants with CHD are less breastfed compared to their healthy peers, both for their clinical conditions and for long periods of mother–infant separation.

Donor breast milk has been shown to be protective against alterations in gut microbiota associated with NEC and feeding intolerance; thus, these benefits may also be considered in infants with CHD [74]. Human milk may be fortified with human milk fortifiers to meet the infant’s metabolic demand. Glucose polymers, medium-chain triglycerides (MCT) oil, and protein supplements can also be added to optimize caloric intake [8]. The use of high-energy formula compared with standard formula in postoperative infants with CHD was associated with weight increase but also increased feeding intolerance [50]. The use of a peptide nutrient-energy dense enteral feed may have particular benefit in infants with cow’s milk protein intolerance [2].

#### 4.3.2. Routes

Infants should always be encouraged to feed orally, if possible. Oral feeding during hospitalization is associated with shorter length of hospital stay, and a decreased risk of neurodevelopmental delay at 12 and 24 months [75]. 

However, several factors, such as fatigue, anorexia, lack of ability in suckling or swallowing, gastroesophageal reflux, may avert this [13]. Oral gastric tube feedings may represent a useful transitional substitute in the short term for providing adequate feed quantity, particularly in neonates with respiratory issues. Alternatively, nasogastric tube feeding should be considered to allow oral feeding training. In cases of delayed gastric emptying or gastroesophageal reflux, a nasojejunal tube may be a valid option too [13].

Therefore, feeding by orogastric or nasogastric tube using either continuous or intermittent bolus delivery of human milk or formula may be provided [76]. Intermittent bolus feeding simulates the feeding pattern of infants when they are breast or bottle fed and has been advocated to promote more physiological feeding–fasting hormonal levels than continuous feedings [76]. Continuous feed has been shown to be a safe and effective way to increase nutrient intake and improve the nutritional status with less energy expenditure than bolus feeds [13]. Each case should be personalized in order to provide an optimal patient-targeted treatment.

Sometimes infants refuse to suck on the breast or feeding bottle, the so-called oral aversion, which is often triggered by oral intubation or surgery [18]. Desensitization of the mouth by an appropriately trained clinician, often a speech therapist, may help facilitate the sucking abilities of the baby. If prolonged nutrition via tube feeding and/or parenteral nutrition is required, when infants have oral aversion or poor coordination of sucking, swallowing, and breathing, a percutaneous endoscopic gastrostomy (PEG) tube may be inserted to maintain growth and development, particularly in those cases undergoing several surgical procedures (such as HLHS) [77]. A structured program with close monitoring of weight and nutritional management may help in the early identification of those cases who may benefit from a PEG [78], sometimes associated with laparoscopic Nissen fundoplication in case of severe gastroesophageal reflux [79].

The diagnosis of a genetic syndrome, single-ventricle physiology, aortic arch reconstruction, and delayed sternal closure were identified as related to gastrostomy tube placement, as well the number of days intubated and the presence of dysphagia [80].

Careful monitoring of nutritional status and growth during hospitalization is recommended as an overall strategy in critically ill patients to improve outcomes [11,12].

#### 4.3.3. Short- and Long-Term Difficulties after Cardiac Surgery

Chylothorax is a known complication of cardiothoracic surgery (up to 5% of cases) [81] and is associated with increased mortality, cost, and length of stay [82]. Conservative management is appropriate as the initial treatment, with a fat-free and MCT-enriched diet [83]. Because of the high level of long-chain triglycerides in human milk, this usually results in the discontinuation of breastfeeding. Modified breast milk that has undergone fat removal, with low fat content (LFBM), has been shown to be an efficient treatment for chylothorax, providing the benefits of human milk equally in those who cannot receive it [84,85]. Furthermore, changes in mean weight, length and head circumference for age Z-scores did not differ among infants nourished with defatted breast milk and infants receiving high-MCT formulas [85].

Given the role of some nerves (vagus, recurrent laryngeal and phrenic) in triggering a timely swallow response and simultaneous airway closure, a post-operative nerve paralysis/paresis could complicate enteral feeding after congenital heart surgery [18]. In a recent review, Sinha et al. reported an incidence of vocal cord palsy ranging from 1.1% to 100% in different studies [86]. Most studies suggest a gradual improvement in swallowing function, whereas the rate of recovery of vocal fold motion impairment is less predictable [87].

Finally, growth failure is common in infants with CHD and has a multifactorial etiology. Mitting et al. described how 28.2% of the infants in their cohort had a mild degree of malnutrition (weight-for-age Z-score < −1 to ≤−2 SDS) and 10.9% had severe malnutrition (<−2 SDS) [88]. A standardized longitudinal monitoring of weight, length and head circumference Z-scores for age allows for earlier identification of failure to thrive, thus improving postoperative outcomes and neurodevelopment [89].

## 5. Conclusions

In this narrative review, we proposed a decisional algorithm that may guide clinicians to optimize growth in infants with CHD, both in the pre- and post-operative period [8,11,78].

However, a multi-disciplinary team is fundamental to improve the nutritional status of CHD infants, as it supports healthcare workers with nutritional standardized protocols and targeted strategies to fight feeding intolerance [90].

Parents and families play a crucial role too. Family support programs can help to address parental concerns and anxieties, increasing confidence and skills to improve their child’s nutritional status and outcome [91,92].

Regular nutritional and growth assessment is essential during inpatient management and during follow-up or home surveillance, and for early intervention in case of failure to thrive.

## Figures and Tables

**Table 1:** NEC alert signs.

“Red Flags”
• Hypotension during inotropes infusion
• Acute respiratory distress
• Apnoea (>2 within 2 h associated to respiratory distress and worsening)
• Disseminated Intravascular Coagulation
• Severe acidosis (pH < 7.15 for >2 h; EB > 10 mmol/L)
• Persistent hypoxia (PaO2 < 40 for >2 h)
• Clinical abdomen signs of suspected NEC
• Bile-colored vomiting or gastric drainage
• Blood in the stool/absence of stool

## Data Availability

Not applicable.
